# Cross-cultural comparisons of aerobic and muscular fitness in Tanzanian and English youth: An allometric approach

**DOI:** 10.1371/journal.pone.0211414

**Published:** 2019-02-15

**Authors:** Joyce Ndabi, Alan M. Nevill, Gavin R. H. Sandercock

**Affiliations:** 1 Department of Physical education and Sports Sciences. University of Dar es Salaam, Dar es Salaam, Tanzania; 2 School of Sport Exercise and Rehabilitation Sciences, University of Essex, Colchester, United Kingdom; 3 Faculty of Education, Health and Wellbeing, University of Wolverhampton, Walsall, United Kingdom; Vanderbilt University, UNITED STATES

## Abstract

Comparisons of physical fitness measures between children or within group measures over time are potentially confounded by differences in body size. We compared measures of strength (handgrip) and aerobic fitness (running-speed [20m shuttle-run]) of 10.0–15.9 year-olds from Dar es Salaam, Tanzania (*n* = 977) with schoolchildren from England (*n* = 1014) matched for age and sex. Differences in fitness were analyzed using general linear models, with allometric scaling for body size (mass and stature) and further adjustments for physical activity. Mean handgrip of Tanzanians was lower than English youth (*F* = 165.0, *P*<0.001, *η*_*p*_^*2*^ = .079). The difference became trivial when run-speed was scaled for body size (*η*_*p*_^*2*^ = .008). Running-speed of the English children was higher than in Tanzanians (*F* = 16.0, *P*<0.001, *η*_*p*_^*2*^ = .014). Allometric scaling for accentuated this between-county difference in running-speed (*η*_*p*_^*2*^ = .019) but when adjusted for physical activity between-country differences in running-speed were trivial (*η*_*p*_^*2*^ = .008). These data contradict those studies showing poor muscular fitness in African youth and highlight the need for appropriate scaling techniques to avoid confounding by differences in body size. In contrast to those from rural areas, our sample of contemporary urban Tanzanians were less aerobically fit than European youth. Differences were independent of body size. Lower aerobic fitness of urban Tanzanian youth may be due to reported physical activity levels lower than those of English youth and lower still than previously reported in rural Tanzania.

## Introduction

Economic growth and urbanization means the historical risks from under-nutrition[1] have been replaced by those from changes in lifestyle indicative of nutritional and physical activity transition[2–4]. Between 1990 and 2010 Dar es Salaam was the third fastest growing urban area in Africa[5] with an annual growth rate of 4.7%[6]. Economic development, population growth and migration have driven rapid urbanization in Dar es Salaam which increased in size from 9 km^2^ in 1945 to become an urban conurbation covering 1000 km^2^ today.[7]

The growing populations of adult Tanzanians who live in urban areas are less physically active and have less-favorable blood-lipid profiles than rural their rural counterparts Tanzanians.[8] Rural to urban migration is associated with reduced physical activity levels and weight gain data in Tanzanian adults.[9]

Comparisons of aerobic fitness provide a consensus that children from sub-Saharan Africa have better aerobic fitness than Europeans of the same age.[10] A comparison of children’s aerobic fitness[11] estimated using the 20 m shuttle-run recently identified Tanzania as the as having the fittest children globally. This claim is based on data from children living in rural upland communities[12] where televisions are a rarity and journeys to school by foot are often many miles daily. In contrast, fewer than 40% of urban Tanzanian children achieve current physical activity recommendations while a quarter report at least three hours daily screen time.

Compared with Europeans, children from rural East Africa are lighter with a lower mass-to-height ratio.[10, 12, 13] Bustamante et al.[14] identified this body shape as optimal for running performance which may account the high values of $\dot{\text{V}}{\text{O}}_{\text{2max}}$ reported when values are estimated using the 20 m shuttle-run[15]. Of the few such comparative studies only one as addressed the potentially confounding effects of differences in body size by scaling for body mass (ml∙kg^-0.75^∙min^-1^). However, $\dot{\text{V}}{\text{O}}_{\text{2max}}$ was however, assessed by cycle ergometry; a non-weight bearing modality which the authors noted was unfamiliar to most rural Tanzanians.

Differences in body size[16] may also explain poorer muscular fitness in African youth when compared with Europeans[10] of the same age. As with differences in running performance, scaling muscular fitness for body size can attenuate[13, 17, 18] or even reverse[15] between-country differences in strength and power.

Few data are available to describe muscular and aerobic fitness of Tanzanian children. To address this gap we measured the aerobic and muscular fitness of children living in Dar Es Salaam; Tanzania’s largest urbanization (commercial city). To assess the influence of body size we compared allometrically scaled muscular and aerobic fitness of Tanzanian and English schoolchildren.

## Methods

The University of Essex Institutional Review Board approved the study and parents gave written informed consent. The study was conducted according to the principles expressed in the Declaration of Helsinki. We adopted the same methodology as the East of England Healthy Hearts Study (EoEHHs) and obtained permission to approach schools from the Ministry of Education and Vocational Training in Dar es Salaam.

### Participants

We used semi-structured convenience sampling to recruit four schools from each of the three municipalities of the Dar es Salaam region. Invitation letters were sent to principals of twelve schools in each district; those who replied were visited by a researcher to explain all procedures and arrange a time for the testing. We structured our sample to include two public schools and two private schools within each region. A sample of *n =* 1028 students from both secondary and primary schools was used.

During the visits the students were informed about all testing procedures and students were given information sheets and letters of informed parental consent. Letters were returned on the day of testing; only those students able to provide evidence of parental consent participated in the assessments. Students without consent continued with their normal curriculum lessons.

Physical activity was measured using the physical activity questionnaire for children (PAQ-C) and adolescents (PAQ-A). These 7-day recall instruments were first translated into Swahili by a native language speaker then independently back-translated. A second translation was then produced and administered to seven student volunteers (aged 11–14 years) fluent in both English and Swahili. On the basis of feedback, minor corrections and amendments were made to produce the final translated questionnaire. All pilot work was completed six weeks before assessments started.

Protocols and equipment used in both countries were identical and all Tanzanian participants were assessed by trained staff from the EoEHHS data collection team. We measured height of the school children to the nearest 1 mm (Seca Leicester Height measure; Seca GmbH & Co. KG, Hamburg, Germany) and weight to the nearest 0.1 kg (Seca 888 digital scale;Seca GmbH & Co.KG). The measurement was taken with participants dressed in in T-shirt and shorts, without shoes. We calculated BMI (mass [kg]/stature [m]^2^) as an index of adiposity and Body Surface (BSA = stature [cm] ∙ mass [kg]/3600)^0.5^ as an estimate of overall body size. Finally, we used the Reciprocal of the Ponderal Index (RPI = stature [m] ∙ mass [kg]^-0.333^) as an index of leanness or ectomorphy.

We measured Handgrip strength of the dominant side using an adjustable analogue dynamometer (T-18 TKK SMEDLY III, Takei Scientific Instruments Co., Ltd, Niigata, Japan). The dynamometer was adjusted according to hand size and researchers demonstrated the correct technique. The highest value recorded from three correctly executed trials was used to indicate maximal handgrip. Aerobic fitness was measured using the FITNESSGRAM PACER. The test requires volunteers to run back and forth at a distance of 20 m in time with an audible signal. The test starts at an initial running speed of 8.0 km∙h^-1^ and increases by 1 km∙h^-1^ after the first minute and then by 0.5 km∙h^-1^ each minute afterwards. The final shuttle count was recorded by the researcher when the participant failed to maintain the required running speed or when the participant has reached their point of volitional exhaustion. Aerobic fitness was expressed as running speed of final stage completed.

We drew a stratified subsample of *n* = 1200 participants from the EoEHHS that matched the Tanzanian data in terms of age and sex frequencies using the case-control matching function in SPSS.

## Statistical analyses

**Fig 1:**
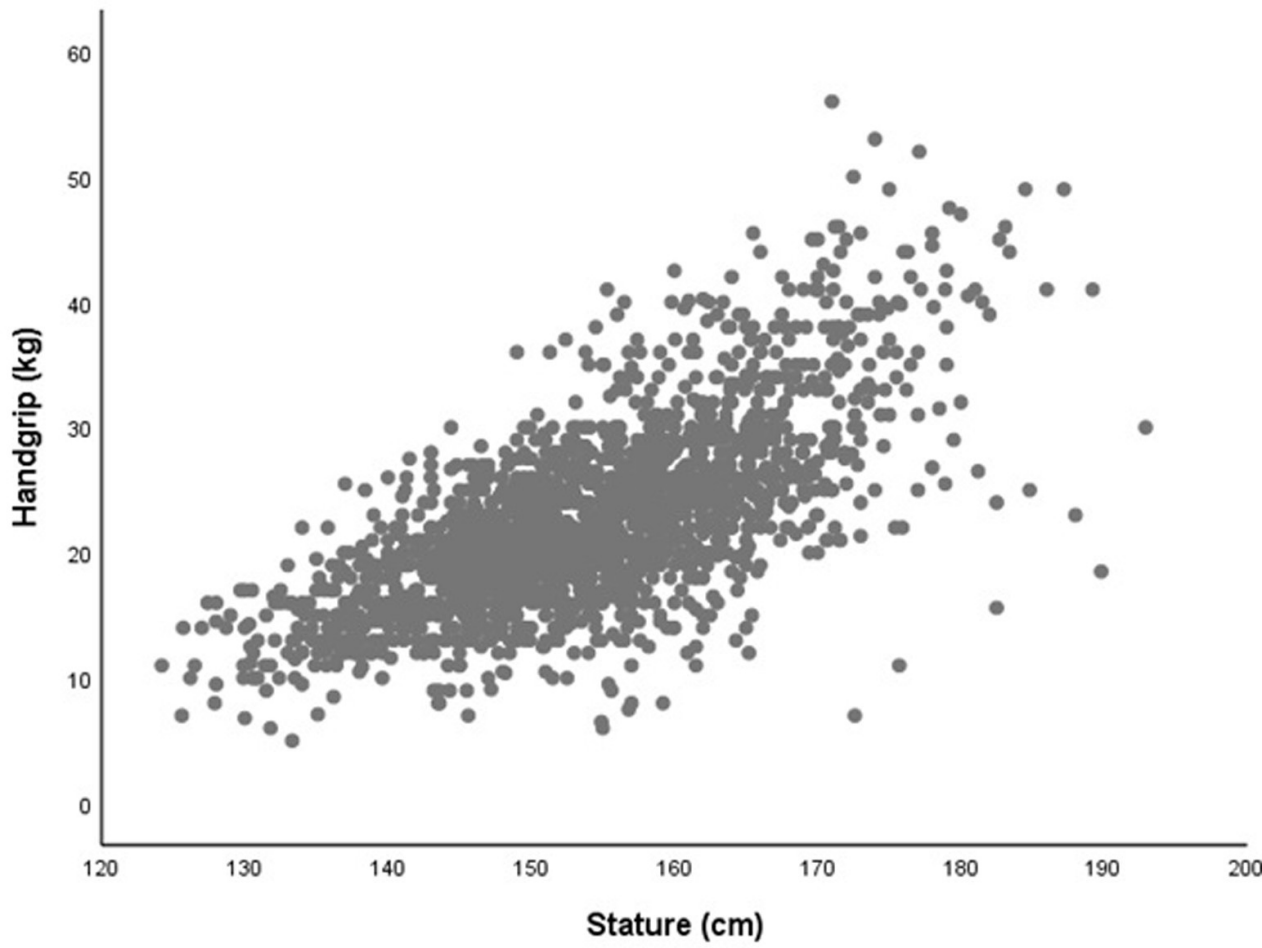
Association of handgrip strength and stature in English and Tanzanian schoolchildren (10.0–15.9 years). Handgrip (kg) assessed as highest value from three trials using the dominant hand. Stature (m) included as an index of overall body size.

**Fig 2:**
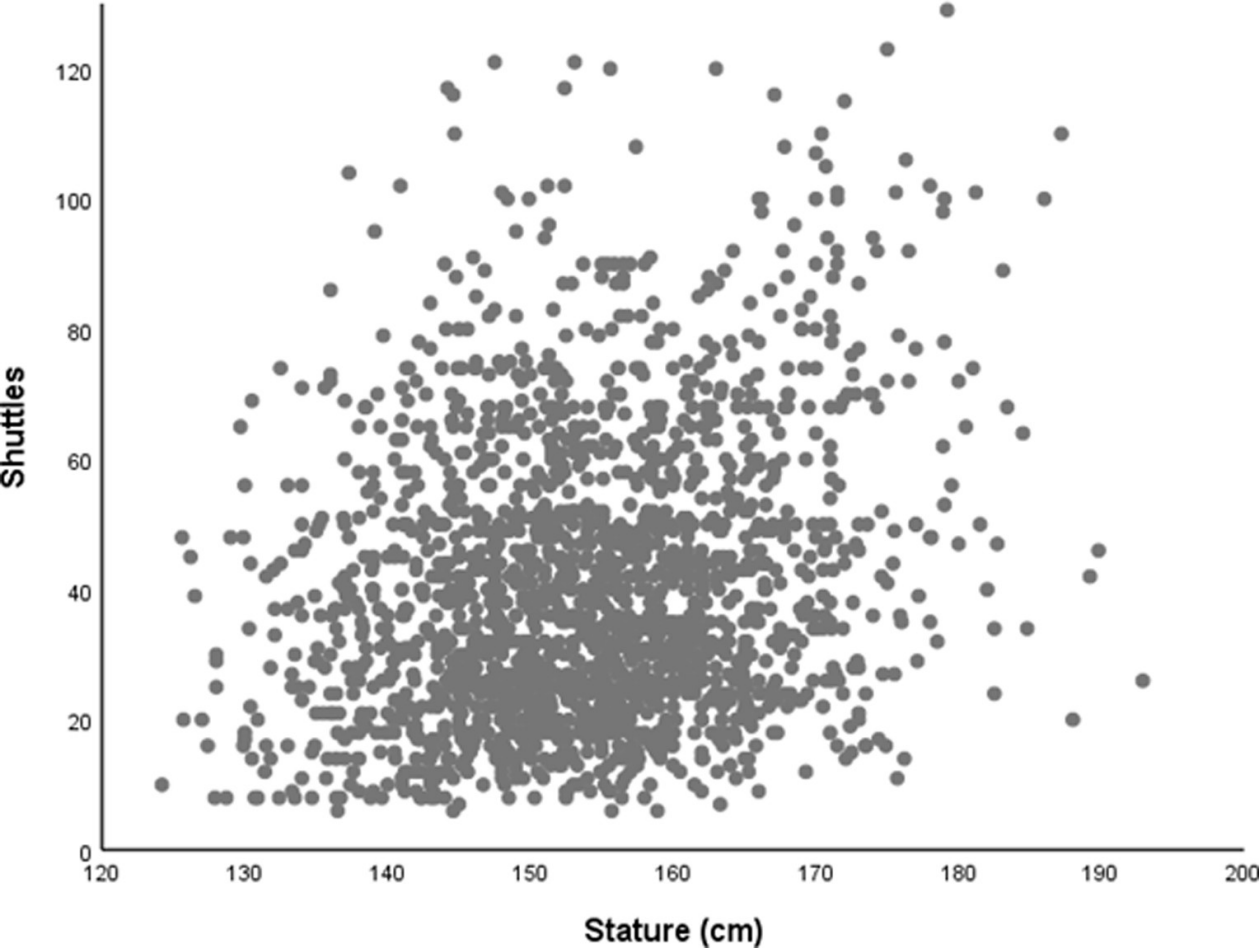
Association of 20 m shuttle-run performance with stature in English and Tanzanian schoolchildren (10.0–15.9 years). Total shuttles shows as a continuous measure of performance on the 20 m shuttle-run test (FITNESSGRAM PACER protocol). Running-speed was derived from total shuttles. Stature (m) included as an index of overall body size.

We calculated means (standard deviations) for anthropometric measures in boys and girls separately and assessed the between-country difference by calculating effect size using Cohen’s *d*. We interpreted values of *d* to indicate; small (*d*>0.2), medium (*d*>0.5) or large (*d*>0.8) effect sizes. We visually inspected scatterplots of outcomes against indices of body size to assess the nature of association. Figs 1 and 2 show the association of stature with handgrip (kg) and running-speed (expressed as total shuttles) respectively. Figs 1 and 2 both show heteroscedascity; with increased error terms at higher measurements values. Handgrip and running-speed can be adjusted for body size allometrically using a multiplicative model with allometric exponents for mass and stature[14, 15, 19–21]. These exponents were derived from general linear models including data from both countries.

The allometric equation Y = a ∙ mass^k1^ ∙ stature^k2^ ∙ ε (where Y is the value of interest and ‘ε’ is the error term) was linearized by taking logs to obtain Ln(Y) = Ln(a) + [*k*_*1*_ ∙ Ln(mass)] + [*k*_*2*_ ∙ Ln(stature)] + Ln(ε). The scaling exponents (*k*_*1*_ and *k*_*2*_) were obtained by including Ln(mass) Ln(stature) as covariates in a general linear model with handgrip or running-speed as the dependent variable Ln(Y). Further categorical or group differences within the population, were assessed for country, sex, age (entered as discrete categories (10 to 15 years) by allowing the constant intercept parameter ‘Ln(a)’ to vary for each group by introducing these categorical variables as fixed factors in the same model.

We calculated the main effects for each categorical variable and reported effect sizes as partial eta squared (*η*_*p*_^*2*^). We interpreted *η*_*p*_^*2*^ values as; small (*η*_*P*_^*2*^>0.01), medium (*η*_*P*_^*2*^>0.05) or large (*η*_*P*_^*2*^>0.14) effect sizes.

## Results

The unadjusted means in Table 1 show that Tanzanian boys and girls had shorter stature and lower body mass compared with English children of the same age. There were medium differences in BSA of Tanzanian and English participants of both sexes. Tanzanians children also had a lower BMI, lower mean values for BSA and RPI. English boys and girls of all ages were more physically active than Tanzanians. There were main effects for age (*F* = 138.5, *P*<0.001, *η*_*p*_^*2*^ = .088), sex (*F* = 138.5, *P*<0.001, *η*_*p*_^*2*^ = .088,) and country (*F* = 138.5, *P*<0.001, *η*_*p*_^*2*^ = .088) and a small age-by-country interaction effect (*F* = 5.0, *P*<0.001, *η*_*p*_^*2*^ = .013).

**Table 1:** Differences in body size, physical fitness and physical activity of 10–15 year olds from England and Tanzania.

		Boys			Girls		
	England		Tanzania		England	Tanzania	
		*n* = *553*	*n* = *476*		*n* = *461*	*n* = *501*	
Stature (cm)	Mean	157	151	*d* = 0.53	155	150	*d* = 0.61
	*SD*	*12*.*1*	*11*.*0*		*8*.*6*	*9*.*9*	
Mass (kg)	Mean	50.1	41.8	*d* = 0.70	49.8	43.7	*d* = 0.54
	*SD*	*13*.*5*	*10*.*1*		*11*.*9*	*10*.*7*	
BMI (kg∙m^-2^)	Mean	20.0	18.1	*d* = 0.54	20.5	19.3	*d* = 0.33
	*SD*	*3*.*5*	*3*.*2*		*3*.*8*	*3*.*6*	
BSA (m^2^)	Mean	2.22	1.87	*d* = 0.70	2.16	1.73	*d* = 0.59
	*SD*	*0*.*73*	*0*.*52*		*0*.*59*	*0*.*57*	
RPI (cm∙kg^-0.333^)	Mean	43.1	43.9	*d* = 0.36	42.7	43.0	*d* = 0.13
	*SD*	*2*.*2*	*2*.*3*		*2*.*4*	*2*.*5*	
Handgrip (kg)	Mean	25.5	22.4	*d* = 0.39	22.2	19.3	*d* = 0.49
	*SD*	*8*.*8*	*7*.*2*		*5*.*7*	*6*.*1*	
Running speed	Mean	10.67	10.38	*d* = 0.26	9.78	9.54	*d* = 0.21
(km∙h^-1^)	*SD*	*1*.*16*	*1*.*16*		*1*.*18*	*1*.*14*	
PAQ-A/C (1–5)	Mean	2.97	2.49	*d* = 0.80	2.67	2.22	*d* = 0.93
	*SD*	0.56	0.64		0.56	0.60	

All values are unadjusted means (standard deviations) Effect sizes calculated as Cohen’s d based on unadjusted means (standard deviations). BSA–Body Surface Area estimated as (stature [cm] ∙ mass[kg])/3600)^0.5^ PAQ-A/C Physical activity questionnaire for children/adolescents (Swahili translation from Voss et al.^29^). Running speed at the final completed stage of the FITNESSGRAM PACER test.

Eq 1 shows the scaling exponents (standard error) for handgrip calculated using all available data. When mass and stature were included as covariates in a general linear model both mass (*F* = 105.4, *P*<0.001) and stature (*F* = 125.1, *P*<0.001) were significant and the scaling exponents were both positive.

$$\text{Handgrip}\;=\;\text{a}\;\cdot {\text{mass}}^{0.325\;(\text{SE}\;0.023)}\cdot \;\;{\text{stature}}^{2.110\;(\text{SE}\;0.113)}$$ (Eq 1)

**Fig 3:**
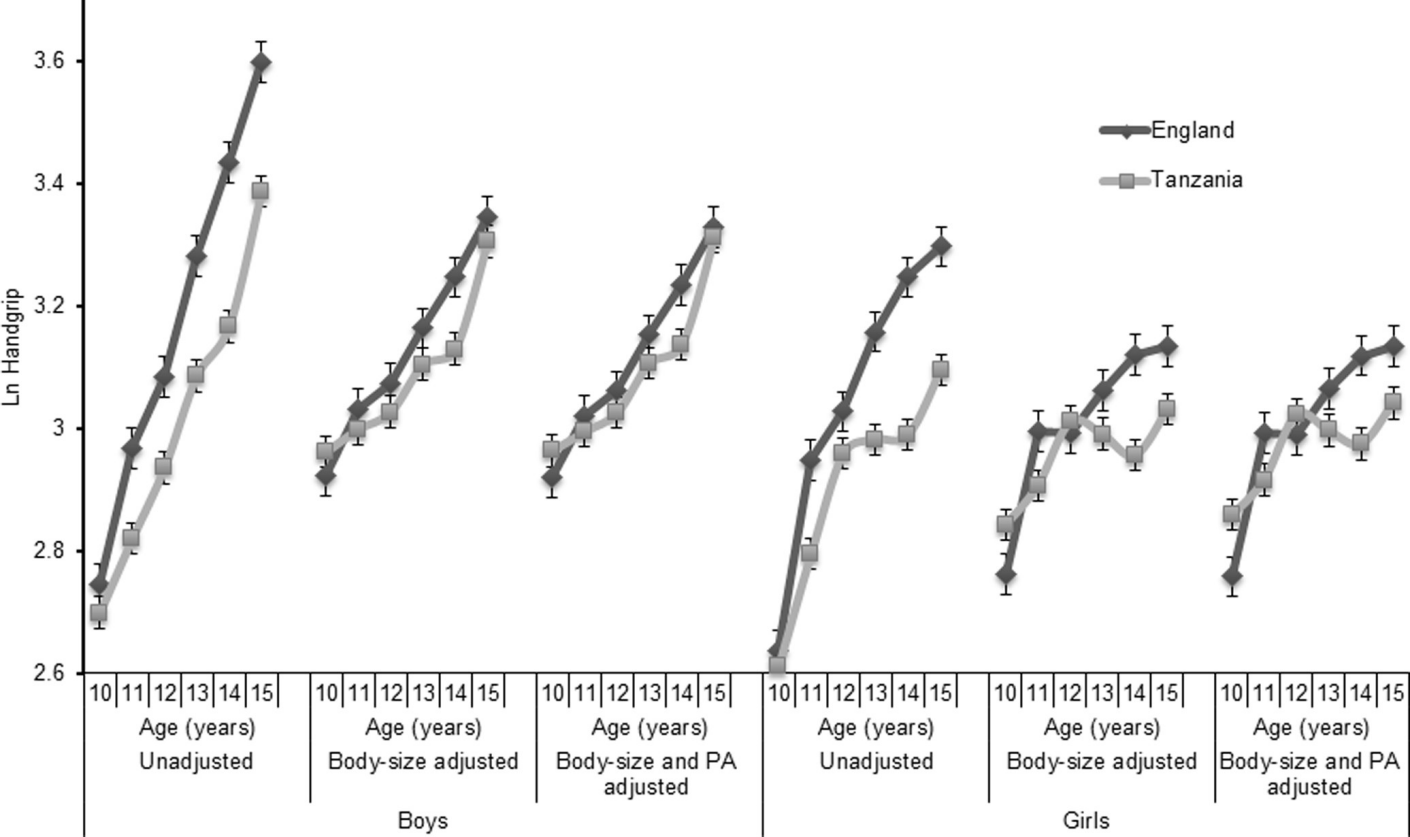
Handgrip strength of English and Tanzanian schoolchildren: Effects of adjusting for body size and physical activity. Body-size adjustments made using allometric scaling for Ln(Mass) and Ln(Stature). PA-Physical Activity–assessed from self-report using the Physical Activity Questionnaire for Children or Adolescents.

Fig 3 shows differences in handgrip between English and Tanzanian children of each sex across all age-groups. For non-adjusted handgrip, there was a large main effect of country (*F* = 165.0, *P*<0.001, *η*_*P*_^*2*^ = 0.079) and a smaller sex-by-country interaction (*F* = 6.2, P<0.008, *η*_*p*_^*2*^ = .015) evident in the divergence of Tanzanian boys’ and girls’ handgrip values after age twelve. Taking the anti-logs of the estimated marginal means across all ages, English children’s handgrip was 3.1 (1.1) kg higher than Tanzanians. The contribution body size makes to differences in handgrip strength is illustrated by the contrast between unadjusted and body-size adjusted models. Adjusting for mass and stature attenuated the main effect for country which remained statistically significant (*F* = 16.5, *P*<0.005) but was trivial in magnitude (*η*_*p*_^*2*^ = .008). The difference in anti-logs of adjusted means was 1.1 (0.6) kg. When introduced to the model PAQ-score was also a significant covariate (*F* = 17.2, *P*<0.001) and further attenuated the main effect of country for handgrip (*F* = 7.8, *P* = 0.005, *η*_*p*_^*2*^ = .004)

**Fig 4:**
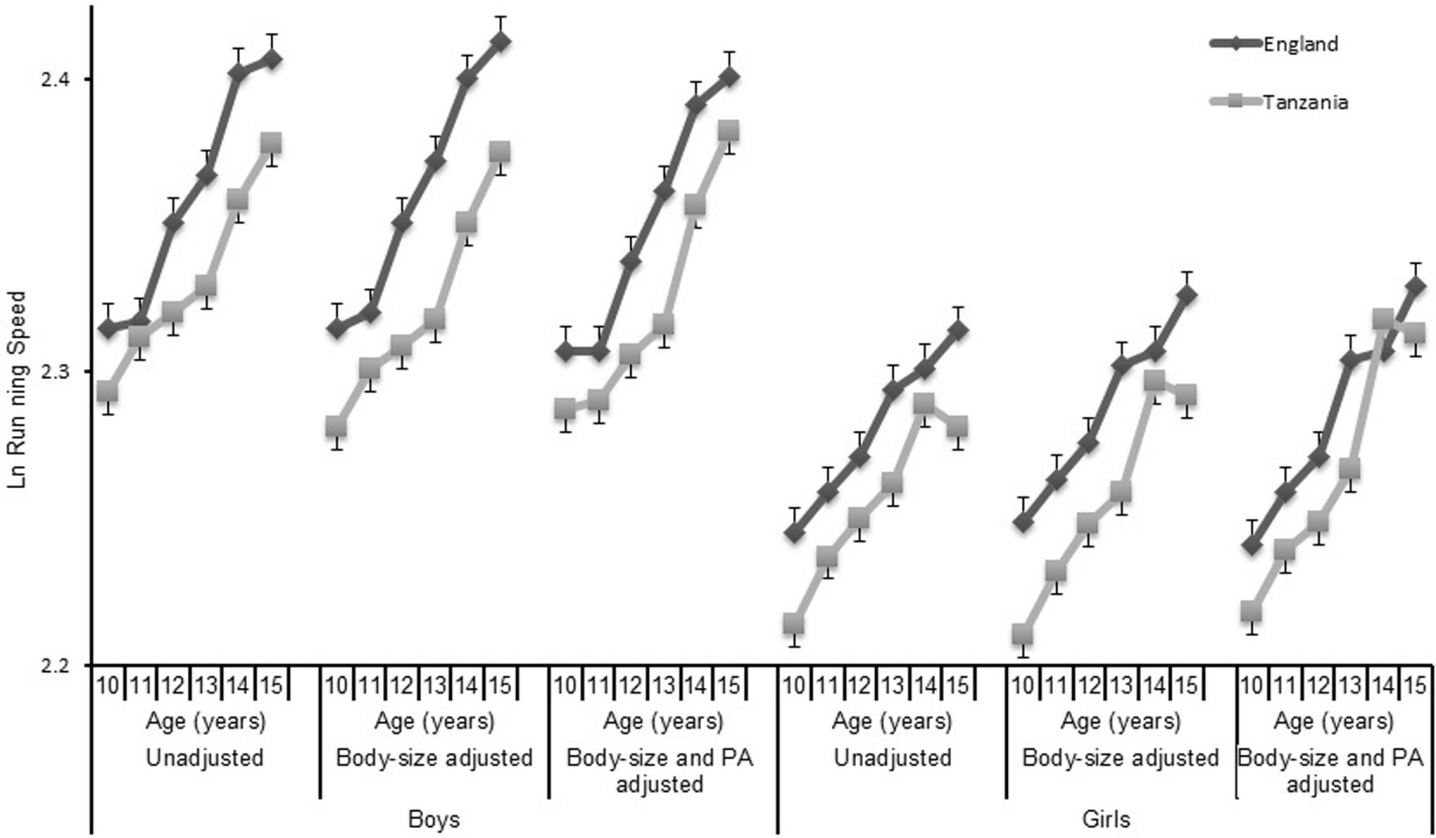
Differences in running speed of English and Tanzanian schoolchildren adjusting for body size and physical activity. Ln—natural logarithm. Running speed is Ln speed (km/h) of last completed stage during the 20 m shuttle-run test. Body-size adjustments made using allometric scaling for Ln(mass) and Ln(stature). PA-Physical Activity–assessed from self-report using the Physical Activity Questionnaire for Children or Adolescents.

Fig 4 shows the estimated marginal means from the three models used to describe differences in running-speed. In the unadjusted model, there was a small main effect for country (*F* = 21.6, *P*<0.001, *η*_*p*_^*2*^ = .012). Tanzanian boys and girls running-speed was, on average, 0.3 km∙h^-1^ slower than that of English children.

$$\text{Running-speed = a}\cdot {\text{mass}}^{-0.157\;\left(\text{SE}\;0.015\right)}\cdot \;\;{\text{stature}}^{0.405\;\left(\text{SE}\;0.059\right).}$$ (Eq 2)

Eq 2 provides the scaling exponents calculated for running-speed. Both factors were significant when introduced as covariates in the general linear model (mass, *F* = 89.0 *P*<0.001) and stature (*F* = 65.4, *P*<0.001) but the exponents were of opposing signs indicating a negative influence of mass but a positive influence of stature.

Adjusting for body size increased the magnitude of the main effect of country for running speed (*F* = 37.5, *P*<0.001, *η*_*p*_^*2*^ = .019). Anti-logs of estimated marginal means revealed English boys (0.4 km∙h^-1^) and girls (0.3 km∙h^-1^) ran faster than Tanzanians. When introduced to the model, PAQ-score was a significant covariate (*F* = 67.4 *P*<0.001) which attenuated the main effect for country. While the difference remained statistically significant, (*F* = 14.9, *P*<0.001) the effect size was trivial (*η*_*p*_^*2*^ = .008). Across all age categories running-speed was marginally slower in Tanzanian boys (0.26 km∙h^-1^) and girls (0.17 km∙h^-1^).

## Discussion

This sample of Tanzanian youth was taken from the rapidly growing urban areas of Dar Es Salaam; a region in nutritional transition[3]. To our knowledge, there are no comparable data describing fitness of contemporary Tanzanian youth from urban areas. Concurrent with findings of studies from across sub-Saharan Africa[10, 12, 15, 17] we found Tanzanian youth children had lower body mass and shorter stature than Europeans of the same age. As differences in body size potentially confound comparisons of fitness between populations[15] we will discuss unadjusted and adjusted values to illustrate the importance of appropriate scaling cross-cultural comparisons of children’s physical fitness.

### Muscular fitness

Handgrip strength increases with children’s age, and in proportion to both their mass and stature[22] as indicated by the positive mass and stature exponents (Eq 1). Compared with Europeans, children in sub-Saharan Africa have lower muscular fitness[10] including poorer handgrip strength.[15, 23] Unadjusted handgrip values of English children were, on average, 13% higher than Tanzanians but as reported previously[17, 23] the European sample were also 5–7% taller and around 20% heavier than their African counterparts.

The low handgrip strength reported in children across sub-Saharan Africa is likely the consequence of reporting absolute values[10] or adjusting only for body mass[23] which does not account for the positive influence of height. This is evident in the positive stature exponent for handgrip found here and reported elsewhere[15, 17, 23]and is probably due to the benefits of greater leverage seen in taller individuals.[22] The importance of employing appropriate scaling in cross-cultural comparisons of fitness was demonstrated by Dos Santos, Nevill (15) who showed that the superior handgrip strength of Portuguese, compared with Mozambican youth was reversed when adjusted for body size, The authors noted that failing to adjust for differences in body size, would have led to the opposite, false conclusion.[15]

Lower adjusted handgrip in 14-year-old boys and older Tanzanian girls could be because European children tend to mature earlier than Africans[13]. This could explain the divergence of handgrip values in older girls and why handgrip of Tanzanian boys’ appears to ‘catch-up’ with that of English 15-year-old. Differences in girls’ handgrip were smaller-still when adjusting for physical activity but the effect was modest compared to adjustments for body size.

### Aerobic fitness

Aandstad *et a*l[12] reported 20 m shuttle-run performance of 9–10 year-old Tanzanians from rural communities in the rural upland area of Mbulu. Mbulu children performed better than Norwegians of that age; the mean running-speed reported for 10-year-olds from Mbulu are faster than any reported here in urban Tanzanians regardless of age and sex.

The low body-weight-to-height ratio evident from the BMI of Mbulu children (14.1 kg∙m^-2^) may have contributed run performance but does not explain why running speed of urban Tanzanians was slower than in English children. BMI valuesof10-year olds inour sample (17.4 kg∙m^-2^) were higher than Mbulu children but lower than English children (18.4 kg∙m^-2^). Despite higher BMI values, English children had faster run speeds than Tanzanians of the same age. Unlike handgrip, adjusting for body size amplified the differences in running-speed further in favor of English children.

These data are not the first showing African children’s fitness is lower than Europeans[12]or in fact, to report low aerobic fitness in urban Tanzanains^8^. Both previous reports may however, be explained by their use of cycle ergometry when estimating fitness. Cycling may be an unfamiliar exercise modality in urban, and particularly rural Tanzanians.[12]

The 20 m shuttle-run speeds reported here are comparable with those of 9-13-year-oldsfrom urban areas of Kenya[23] but slower than children from rural villages. While the authors[23] suggested the urban landscape negatively influenced fitness of Kenyan children but the urban-rural comparisons reported were not adjusted for differences in body size and higher levels of adiposity seen in urban youth. The low height-weight ratio of urban body shape is the ideal for promoting optimal running performance in youth; differences in body size make comparisons of running performance impossible without adequate adjustments for the influence of body size.

Dos Santos *et al*.[15] reported faster running speeds for Portuguese children compared with youth from Mozambique but unlike these authors[15]we did not find differences to be attenuated when adjusting for body size. Instead, differences were amplified, likely because the more favorable body dimensions of Tanzanians.[15, 24, 25] Prista et al.[26] highlighted the importance of physical activity to the development of aerobic fitness in Mozambique youth. The superior aerobic fitness of rural children has also been attributed to a lifestyle that is more physically active than that of urban dwellers.

There is strong evidence that traditional forms of physical activity such as active commuting and household chores have declined in Tanzanian population.[2–4] While overall PA was much lower in Tanzanians we cannot rule out the possibility that our questionnaire failed to detect these types of activity as the PAQ-A/C both focus on sporting and leisure-time activities[27]. Regardless of possible under-reporting, the physical activities undertaken by Tanzanian youth do not seem to be promoting good aerobic or muscular fitness in this population.

Another reason that physical activity scores differed consistently between Tanzanian schoolchildren and those from England is due to differences in provision of Physical Education (PE). Provision of school PE is associated with manifold physical and psychological benefits[28]. At the time of testing all English children received 2-hours of curriculum-time PE each week and were offered an additional two hours school sport or activity. In Tanzania at the time of testing by contrast, PE was not a mandated subject in public schools and often did not appear on the curriculum of the private schools. Where offered, PE is, instead, regarded as an extracurricular activity taken on a voluntary basis after-school.

### Limitations

The limitations of self-reported PA are well documented but the may be compounded in the present study because the instrument used was originally designed and validated for use in North American youth[27]. Notwithstanding the problems associated with translation and interpretation, the cultural norms which informed its design may have artificially lowered Tanzanian children’s scores. First, one item assumes children receive physical education as is mandatory as many children did not receive PE lessons their responses would have been invalid. Second, the Anglicized version of the PAQ-A/C used in the EoEHHS was modified to include traditional sports (such as cricket and rounders)[29] but we were unable to modify the list again for use in Tanzania. Most importantly, the PAQ does not assess school transport or household tasks. It is likely that Tanzanian children may have spent more time engaged in these activities than English or North American youth.

## Conclusion

These data present a challenge to the consensus that handgrip strength of African children is inferior to that of Europeans and highlight the need for appropriate scaling when comparing fitness between groups of different body size [15, 20, 22, 25].

The aerobic fitness of this contemporary sample of urban Tanzanian children is much lower than that previously reported in rural populations. Urban Tanzanians are less active than English children and body size low activity seems the most likely cause of low fitness. Future research should investigate effects of urbanization on the physical activity and fitness in Tanzanian youth but such studies must use appropriate scaling.

## References

[1] Jinabhai CC, Taylor M, Sullivan KR. Implications of the prevalence of stunting, overweight and obesity amongst South African primary school children: a possible nutritional transition? Eur J Clin Nutr. 2003;57(2):358–65. 10.1038/sj.ejcn.1601534 . 12571672

[2] Maletnlema TN. A Tanzanian perspective on the nutrition transition and its implications for health. Public Health Nutr. 2002;5(1A):163–8. . 12027280 10.1079/phn2001289

[3] Keding G. Nutrition Transition in Rural Tanzania and Kenya. Hidden Hunger: Malnutrition and the First 1,000 Days of Life: Causes, Consequences and Solutions. 2016;115:68–81. 10.1159/000442073 PubMed PMID: WOS:000376934200009. 27198465

[4] Keding GB, Msuya JM, Maass BL, Krawinkel MB. Dietary patterns and nutritional health of women: The nutrition transition in rural Tanzania. Food Nutr Bull. 2011;32(3):218–26. PubMed PMID: WOS:000295861100006. 10.1177/156482651103200306 22073796

[5] Hill A, Lidner C. Land-use modelling to support strategic urban planning—the case of Dar es Salaam, Tanzania 45th ISOCARP Congress 2010. 2010.

[6] Forum WE. These are Africa’s fastest-growing cities. World Economic Forum, 2015.

[7] Mkalawa CC, Haixiao P. Dar es Salaam city temporal growth and its influence on transportation. Urban, Planning and Transport Research. 2014;2(1):423–46. 10.1080/21650020.2014.978951

[8] Mbalilaki JA, Hellenius ML, Masesa Z, Hostmark AT, Sundquist J, Stromme SB. Physical activity and blood lipids in rural and urban Tanzanians. Nutr Metab Cardiovasc Dis. 2007;17(5):344–8. 10.1016/j.numecd.2006.03.003 . 17134959

[9] Unwin N, James P, McLarty D, Machybia H, Nkulila P, Tamin B, et al Rural to urban migration and changes in cardiovascular risk factors in Tanzania: a prospective cohort study. BMC Public Health. 2010;10:272 10.1186/1471-2458-10-272 20497567 PMC2892446

[10] Muthuri SK, Wachira LJ, Leblanc AG, Francis CE, Sampson M, Onywera VO, et al Temporal trends and correlates of physical activity, sedentary behaviour, and physical fitness among school-aged children in Sub-Saharan Africa: a systematic review. Int J Environ Res Public Health. 2014;11(3):3327–59. 10.3390/ijerph110303327 24658411 PMC3987037

[11] Lang JJ, Tremblay MS, Leger L, Olds T, Tomkinson GR. International variability in 20 m shuttle run performance in children and youth: who are the fittest from a 50-country comparison? A systematic literature review with pooling of aggregate results. Br J Sports Med. 2016 10.1136/bjsports-2016-096224 . 27650256

[12] Aandstad A, Berntsen S, Hageberg R, Klasson-Heggebø L, Anderssen S. A comparison of estimated maximal oxygen uptake in 9 and 10 year old schoolchildren in Tanzania and Norway. Br J Sports Med. 2006;40(4):287–92. 10.1136/bjsm.2005.020040 16556780 PMC2577514

[13] Prista A, Maia JAR, Damasceno A, Beunen G. Anthropometric indicators of nutritional status: implications for fitness, activity, and health in school-age children and adolescents from Maputo, Mozambique. Am J Clin Nutr. 2003;77(4):952–9. PubMed PMID: WOS:000181747600031. 10.1093/ajcn/77.4.952 12663297

[14] Bustamante VA, Maia J, Nevill A. Identifying the ideal body size and shape characteristics associated with children's physical performance tests in Peru. Scand J Med Sci Sports. 2015;25(2):e155–65. 10.1111/sms.12231 . 24779794

[15] Dos Santos FK, Nevill A, Gomes TN, Chaves R, Daca T, Madeira A, et al Differences in motor performance between children and adolescents in Mozambique and Portugal: impact of allometric scaling. Ann Hum Biol. 2016;43(3):191–200. 10.3109/03014460.2015.1024738 . 26207594

[16] Jurimae T, Hurbo T, Jurimae J. Relationship of handgrip strength with anthropometric and body composition variables in prepubertal children. Homo. 2009;60(3):225–38. 10.1016/j.jchb.2008.05.004 . 18996520

[17] Heroux M, Onywera V, Tremblay MS, Adamo KB, Lopez Taylor J, Jauregui Ulloa E, et al The Relation between Aerobic Fitness, Muscular Fitness, and Obesity in Children from Three Countries at Different Stages of the Physical Activity Transition. ISRN Obes. 2013;2013:134835 10.1155/2013/134835 24533216 PMC3901969

[18] Malina RM, Little BB, Shoup RF, Buschang PH. Adaptive significance of small body size: Strength and motor performance of school children in Mexico and Papua New Guinea. Am J Phys Anthropol. 1987;73(4):489–99. 10.1002/ajpa.1330730411 3661686

[19] Nevill AM, Holder RL, Baxter-Jones A, Round JM, Jones DA. Modeling developmental changes in strength and aerobic power in children. J Appl Physiol (1985). 1998;84(3):963–70. 10.1152/jappl.1998.84.3.963 . 9480958

[20] Jaric S. Role of body size in the relation between muscle strength and movement performance. Exerc Sport Sci Rev. 2003;31(1):8–12. 10.1097/00003677-200301000-00003 PubMed PMID: WOS:000180596000003. 12562164

[21] Nevill AM, Bate S, Holder RL. Modeling physiological and anthropometric variables known to vary with body size and other confounding variables. Yearbook of Physical Anthropology, Vol 48. 2005;48:141–53. 10.1002/ajpa.20356 PubMed PMID: WOS:000235159600007. 16369959

[22] Nevill A, Tsiotra G, Tsimeas P, Koutedakis Y. Allometric associations between body size, shape, and physical performance of Greek children. Pediatr Exerc Sci. 2009;21(2):220–32. . 19556627 10.1123/pes.21.2.220

[23] Adamo KB, Sheel AW, Onywera V, Waudo J, Boit M, Tremblay MS. Child obesity and fitness levels among Kenyan and Canadian children from urban and rural environments: A KIDS‐CAN Research Alliance Study. Int J Pediatr Obes. 2011;6(2Part2):e225–e32. 21198357 10.3109/17477166.2010.543683

[24] Nevill AM, Markovic G, Vucetic V, Holder R. Can greater muscularity in larger individuals resolve the 3/4 power-law controversy when modelling maximum oxygen uptake? Ann Hum Biol. 2004;31(4):436–45. 10.1080/03014460410001723996 PubMed PMID: WOS:000224533900006. 15513694

[25] Silva S, Bustamante A, Nevill A, Katzmarzyk PT, Freitas D, Prista A, et al An Allometric Modelling Approach to Identify the Optimal Body Shape Associated with, and Differences between Brazilian and Peruvian Youth Motor Performance. PLoS One. 2016;11(3):e0149493 10.1371/journal.pone.0149493 26939118 PMC4777497

[26] Prista A, Nhantumbo L, Saranga S, Lopes V, Maia J, Vinagre J, et al Physical activity assessed by accelerometry in rural African school-age children and adolescents. Pediatr Exerc Sci. 2009;21(4). 10.1123/pes.21.4.384 20128359

[27] Kowalski KC, Crocker P, Kowalski NP. Convergent validity of the Physical Activitiy Questionnaire for Adolescents. Ped Exerc Res. 1997;9:342–52.

[28] Fairclough S, Stratton G. 'Physical education makes you fit and healthy'. Physical education's contribution to young people's physical activity levels. Health Educ Res. 2005;20(1):14–23. 10.1093/her/cyg101 . 15253994

[29] Voss C, Ogunleye AA, Sandercock GR. Physical Activity Questionnaire for Children and Adolescents: English norms and cut-points. Pediatr Int. 2013;55(4):498–507. 10.1111/ped.12092 . 23461812

